# Morphologic and Morphometric Study of the Heart and Its Great Arteries in the Common Pheasant (*Phasianus colchicus*)

**DOI:** 10.1002/vms3.70806

**Published:** 2026-01-14

**Authors:** Hadis Ramezani, Nader Goodarzi

**Affiliations:** ^1^ Faculty of Veterinary Medicine Razi University Kermanshah Iran; ^2^ Department of Basic Sciences and Pathobiology Faculty of Veterinary Medicine Razi University Kermanshah Iran

**Keywords:** avian, cardiovascular system, common pheasant, desmin, α‐SMA

## Abstract

This study investigates the morphological and morphometric characteristics of the heart and great arteries in the common pheasant (*Phasianus colchicus*). Five adult male bird were used. Different morphometric and histomorphometric parameters in the heart and its great arteries were measured. Detailed structure of the heart chambers was investigated using scanning electron microscope (SEM). The expression pattern of desmin α‐smooth muscle actin (α‐SMA) was evaluated by immunohistochemical staining. The heart was elongated and conical heart with mean length and width as 2.94 ± 0.46 and 2.38 ± 0.15 cm, respectively. The parietal wall of the right ventricle was composed of two distinct muscular layers. The left ventricular wall at middle and apical regions was thicker than right ventricular wall four and three times, respectively. At the level of SEM, the right muscular atrioventricular valve was attached to the right ventricular free wall by several muscular cords. The chordae tendineae of the left atrioventricular valve showed a branched appearance and each chordae tendineae was composed of three to four narrower cords twisted to each other's and attached to a common papillary muscle. The Purkinje fibre network was widely distributed in the myocardium and exhibited strong immunoreactivity for desmin but was negative for α‐ α‐SMA. In conclusion, the consistent morphological and immunohistochemical patterns observed across individuals provide a reliable description of cardiovascular adaptations in the common pheasant. The results contribute to the broader understanding of avian heart morphology and function, offering a foundation for comparative studies across bird species and informing conservation efforts for gamebirds.

## Graphical abstract

The common pheasant heart is elongated and conical, with thicker left ventricular walls and a complex atrioventricular valve structure.

Purkinje fibres show strong desmin but no α‐SMA expression, providing detailed insights into cardiovascular morphology and adaptations in birds, supporting comparative and conservation studies.

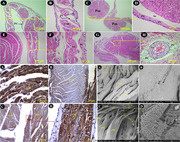

## 1 Introduction

The avian cardiovascular system exhibits unique structural adaptations that support the high metabolic demands of flight, including a fully separated four‐chambered heart and an efficient closed circulatory system. In birds, the heart is typically larger relative to body size than in other vertebrates, reflecting the elevated oxygen requirements associated with powered flight (Straub et al. 2002). The right and left ventricles also display clear structural differences: the right ventricle is thinner and more compliant to accommodate the lower‐pressure pulmonary circulation, whereas the left ventricle has a thicker wall to overcome systemic vascular resistance. These adaptations contribute to efficient oxygen delivery and dynamic regulation of blood flow during various physiological states such as rest, activity and flight.

The morphology and morphometry of the avian heart have been examined in numerous species at both macroscopic and microscopic levels, providing important insights into cardiovascular specialization in birds (Al Masri et al. 2017; Hassanzadeh et al. 2005; Anatskaya and Vinogradov 2002; Olkowski et al. 1999; Harash et al. 2019; V. Prosheva et al. 2019; Kharin et al. 2006; Elewa et al. 2018; V. I. Prosheva and Kaseva 2016). Studies on budgerigars, Alisterus parrots, common buzzards (Straub et al. 2002) and *Caracara plancus* (Miguel et al. 2014) have established reference values for myocardial thickness and ventricular dimensions. Investigations in the greater rhea have shown significant differences in heart dimensions and ventricular wall thickness between young and adult birds (Borges et al. 2021), supporting the idea that the avian heart undergoes structural and functional adaptation throughout development.

The common pheasant (*Phasianus colchicus*) is a widely distributed gamebird known for its distinctive plumage, ecological flexibility and complex social behaviour (Kayvanfar et al. 2015). Despite extensive research on its external morphology and ecology, detailed anatomical and histological studies of its cardiovascular system remain limited. Understanding the cardiac morphology of this species is important not only for avian anatomical knowledge but also for evaluating factors that influence fitness, survival and physiological performance.

Therefore, the present study aims to investigate the morphological and morphometric characteristics of the heart and major arteries in the common pheasant. By providing comprehensive gross, histological, ultrastructural and immunohistochemical descriptions, this research contributes valuable information for comparative cardiovascular studies and may support conservation programs for gamebird populations.

## 2 Materials and Methods

### 2.1 Samples Collection and Preparation

In the present study, five adult male common pheasants with an average body weight of 2700 ± 215 g were obtained from breeding farms in Rasht, northern Iran. The birds were transported to the dissection room and humanely euthanized by intramuscular administration of ketamine (10 mg/kg bodyweight), followed by cervical dislocation. Immediately afterward, the thoracoabdominal cavity was opened, and the heart along with its major arteries was carefully exposed and dissected.

### 2.2 Morphometric Measurements

The excised hearts were first weighed using a digital laboratory balance, and the relative heart mass was calculated using the following formula:

Relative heart mass: heart mass / body mass × 100

**FIGURE 1:**
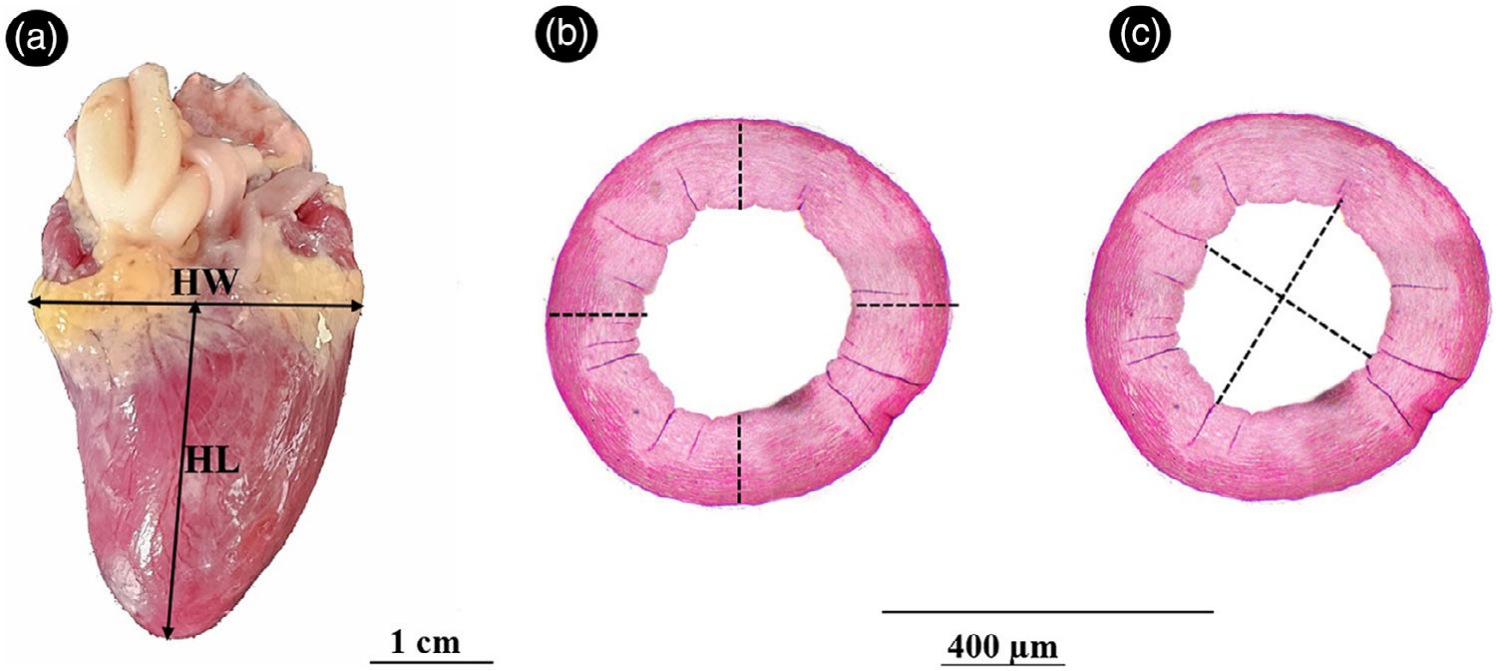
Location of different morphometric and histometric measurements in the common pheasant heart. (A) Position of measuring the heart width (HW) and heart length (HL), (B) cross section of aortic wall for measuring the wall thickness in four different sites, (C) cross section of aortic wall for measuring the luminal diameter.

Then the following morphometric parameters were measured using a Vernier calliper (200 mm; Mitutoyo Corp., Kawasaki, Japan; resolution 0.05 mm, graduation 0.05 mm, accuracy ± 0.05 mm):

− Length of the heart (from the apex to the roots of great vessels at the base).
− Width of the heart (from widest level of the heart, Figure 1A).
− Diameter of the aorta
− Diameter of the left and right pulmonary arteries
− Diameters of the right and left brachiocephalic trunk

### 2.3 Scanning Electron Microscope Study

For SEM analysis, one formalin‐fixed heart was dissected to expose the aorta and pulmonary artery. Small tissue pieces from the auricles, ventricles, atria and atrioventricular valves were collected and dehydrated through ascending ethanol concentrations (50%, 70%, 90% and 100%). Samples were then dried in a freeze dryer, mounted on aluminium stubs and coated with gold. The specimens were examined under a scanning electron microscope (Quanta 450, FEI, USA) at an accelerating voltage of 15 kV.

### 2.4 Histometric Measurements

Four heart samples were fixed in 10% buffered formalin. After 5 days, they were dehydrated in an ascending ethanol series, cleared in xylene and embedded in paraffin. Paraffin blocks were sectioned at 5 μ using a rotary microtome. Sections were stained with haematoxylin and eosin (H&E) and Masson's trichrome.

Using an optical microscope (Olympus CX2, Japan) equipped with a digital camera (KECAM, 5 MP), images were captured at 40× and 100× magnification. Images were analysed using ImageJ software, calibrated before measurement. The following parameters were assessed:

- Thickness of the aortic wall and pulmonary artery wall (measured at four locations and averaged)
- Luminal diameter of the aorta, brachiocephalic trunks and pulmonary arteries (mean of the largest horizontal and vertical diameters; Figure 1B,C)
- Luminal area of the aorta, brachiocephalic trunks and pulmonary arteries (measured using freehand selection in ImageJ)
- Thickness of the left ventricular wall (LVW), right ventricular wall (RVW) and interventricular septum (IVS) in basal, middle and apical regions
- LVW:RVW thickness ratio

### 2.5 Immunohistochemical Study

Immunohistochemical staining was performed on 5‐µm paraffin sections. Sections were deparaffinized, rehydrated and subjected to heat‐induced antigen retrieval in citrate buffer (pH 6.0) at 95°C for 24 min. Endogenous peroxidase activity was quenched using 3% hydrogen peroxide for 20 min at room temperature. After washing in TBS‐Tween, sections were incubated overnight at 4°C with the following primary antibodies: anti‐desmin rabbit polyclonal antibody (ab15200; 1:200), anti‐α‐SMA rabbit polyclonal antibody (ab5694; 1:200) and anti‐S100 mouse monoclonal antibody (ab4066; 1:50). Negative controls were prepared by omitting the primary antibody. Following PBS washes, slides were incubated with a biotinylated secondary antibody and streptavidin‐HRP (30 min each), and immunoreactivity was visualized using DAB (Sigma). Sections were counterstained with haematoxylin, dehydrated and mounted for microscopic examination.

The slides were examined and photographed at 100× and 400× magnifications with an optical microscope (Olympus, Japan; Olympus BX50) connected to the camera system (KEcam Technologies, 5 MP).

### 2.6 Statistical Analysis

Data are presented as mean ± standard deviation (SD). Statistical analysis was performed using SPSS Statistics 17.0. Comparisons were made using the *t*‐test, and a *p*‐value < 0.05 was considered statistically significant.

## 3 Results

### 3.1 Gross Morphology

**FIGURE 2:**
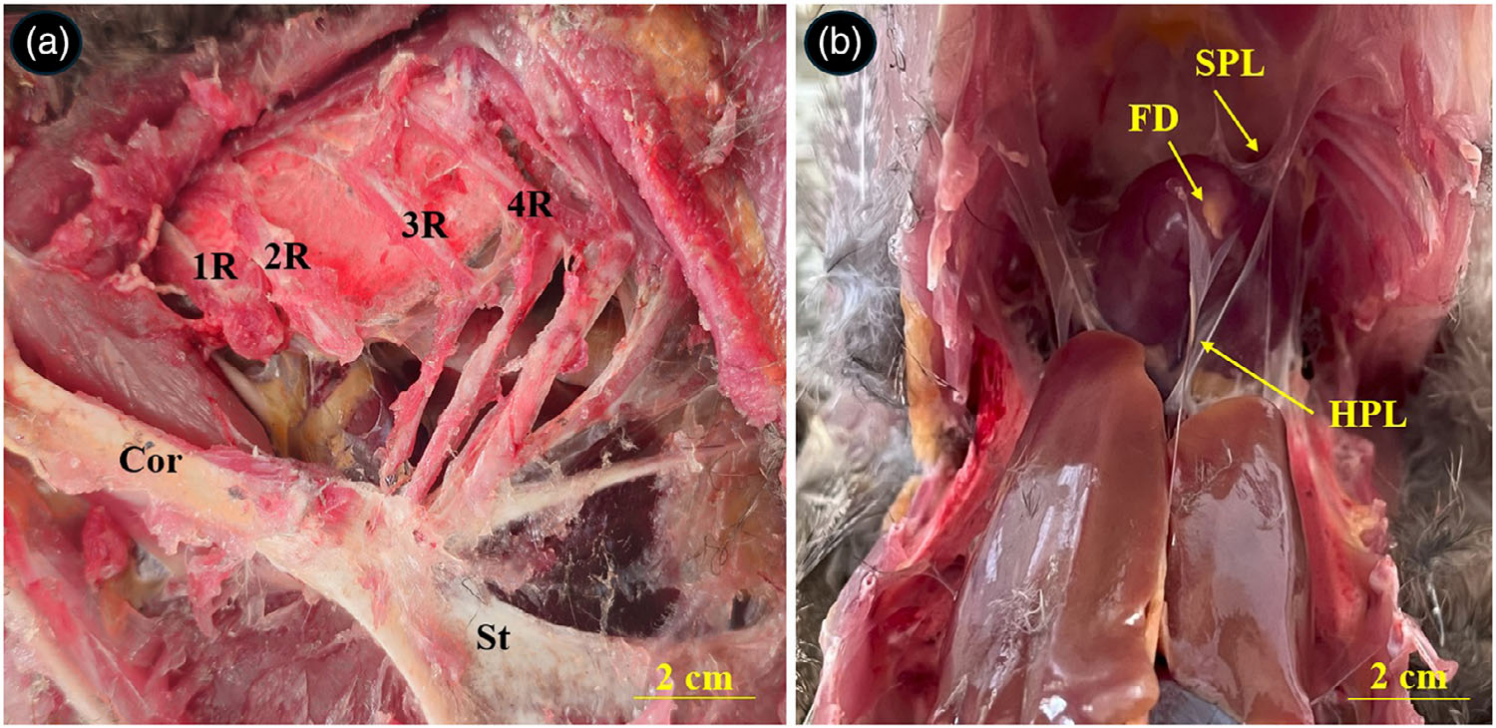
Topography of the heart and its attachments in the thoracoabdominal cavity of the common pheasant. (A) Shows topographic position of the heart between the first and fourth ribs, (B) shows the heart attached to the liver and sternum by hepato‐pericardial ligament (HP) and sterno‐pericardial ligament (SPL), respectively and fat deposit on the heart apex. R: rib, ST: sternum, Cor: coracoid.

**FIGURE 3:**
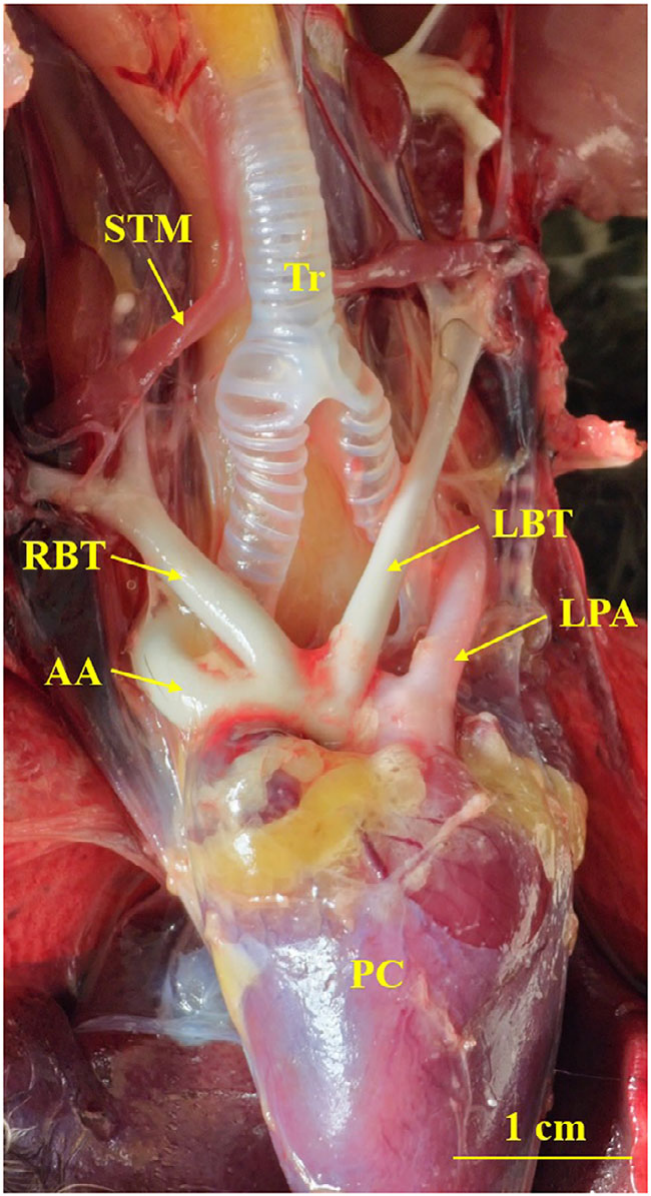
Gross appearance of the great arteries at the base of the common pheasant heart. STM: sterno‐tracheal muscle, RBT: right brachiocephalic trunk, LBT: left brachiocephalic trunk, AA: ascending aorta, LPA: left pulmonary artery, Tr: trachea.

**FIGURE 4:**
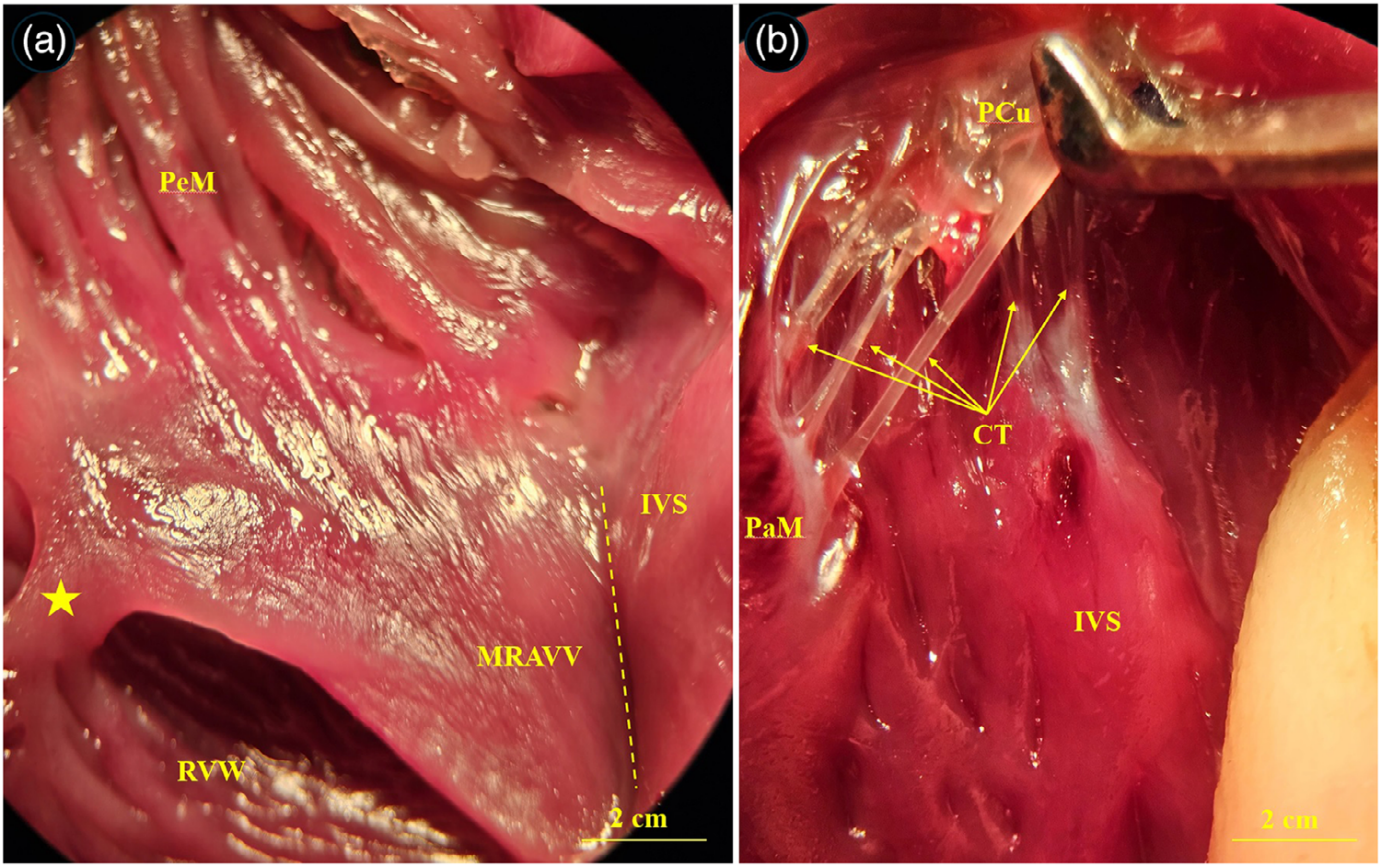
Gross appearance of the atrioventricular valves in the common pheasant heart. (A) Right atrioventricular valve, (B) left atrioventricular valve. PeM: pectinate muscle, IVS: interventricular septum, MRAVV: muscular right atrioventricular valve, RVW: right ventricular wall, CT: chordae tendineae, PCu: parietal cusp, asterisk: parietal attachment of the MRAVV to the right ventricular wall, Dashed line: attachment of the MRAVV to the IVS.

**FIGURE 5:**
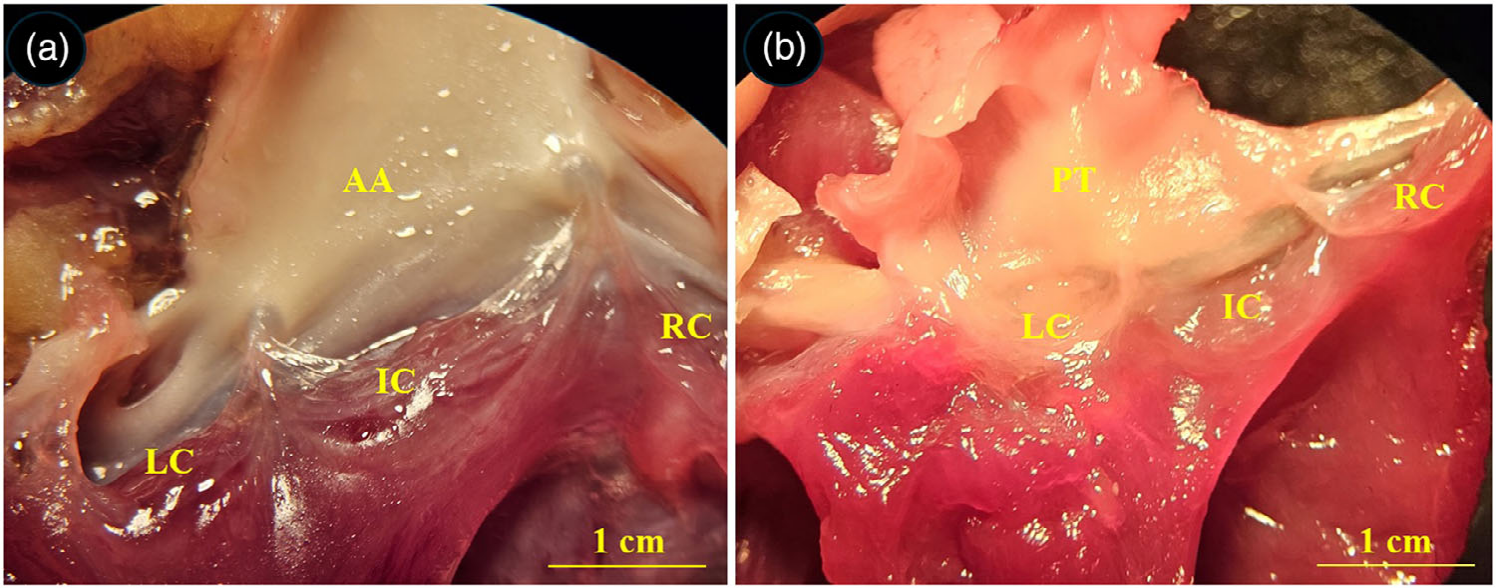
Gross appearance of the semilunar valves in the common pheasant heart. (A) aortic semilunar valve, (B) pulmonary semilunar valve. AA: ascending aorta, PT: pulmonary trunk, LC: left cusp, IC: intermediate cusp, RC: right cusp.

The heart of the common pheasant had an elongated conical shape with blunt apex. Its cranial border was convex, and the caudal border was slightly concave. The base of the heart was lied against the first rib and the heart apex reached under the fourth rib (Figure 2A). The heart was attached to the sternum and liver by sterno‐pericardial and hepato‐pericardial ligaments, respectively. The apex of the heart contained a fat deposit (Figure 2B). An aortic arch and pulmonary trunk emerged from the base of the heart. The aortic arch divided into two left and right brachiocephalic trunks. The pulmonary trunk was also raised to the right and left pulmonary arteries (Figure 3). The muscular right atrioventricular valve (MRAVV) was attached to the free wall of the right ventricle by one attachment and joined to the IVS by a vertical attachment. The RAVV had a muscular structure and no chordae tendineae was present (Figure 4A). The left atrioventricular valve consisted of three leaflets each were attached to the papillary muscles by two to four chordae tendineae (Figure 4B). The aortic and pulmonary valves consisted of three semilunar cusps (Figure 5).

### 3.2 SEM Observations

**FIGURE 6:**
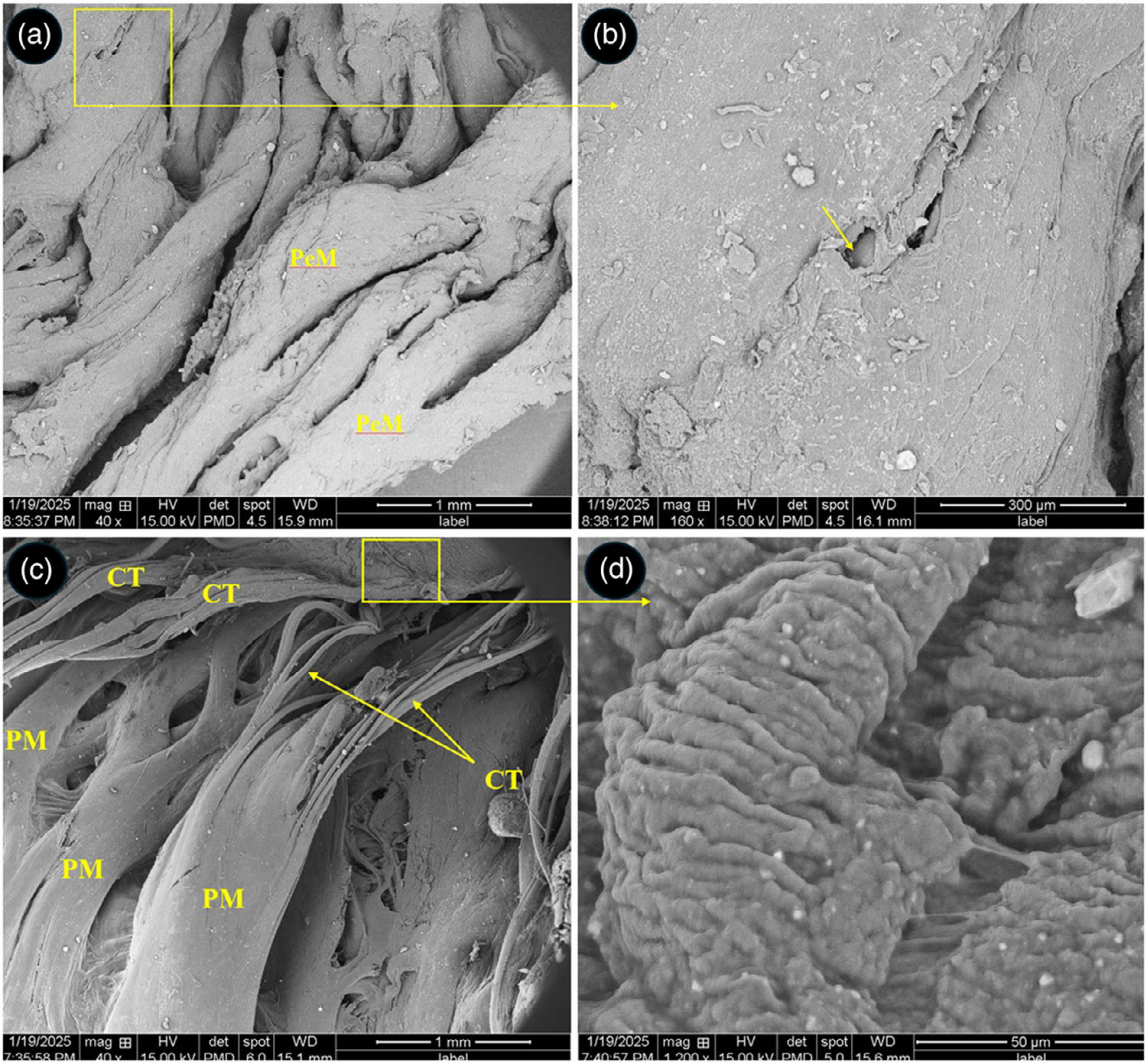
SEM micrograph of the right auricle of the right atrium and left atrioventricular valve in the common pheasant heart. (A) shows several pectinate muscles (PeM) arranged parallel to each other's, (B) shows a fenestrate (arrow) between pectinate muscles at higher magnification, (C) shows chordae tendineae (CT) of the left atrioventricular valve. Note to the branched appearance of each chordae tendineae which composed of narrower cords and attached to a common papillary muscle (PM), (D) shows the selected box in C at higher magnification. The surface of valvar cups has folded appearance.

**FIGURE 7:**
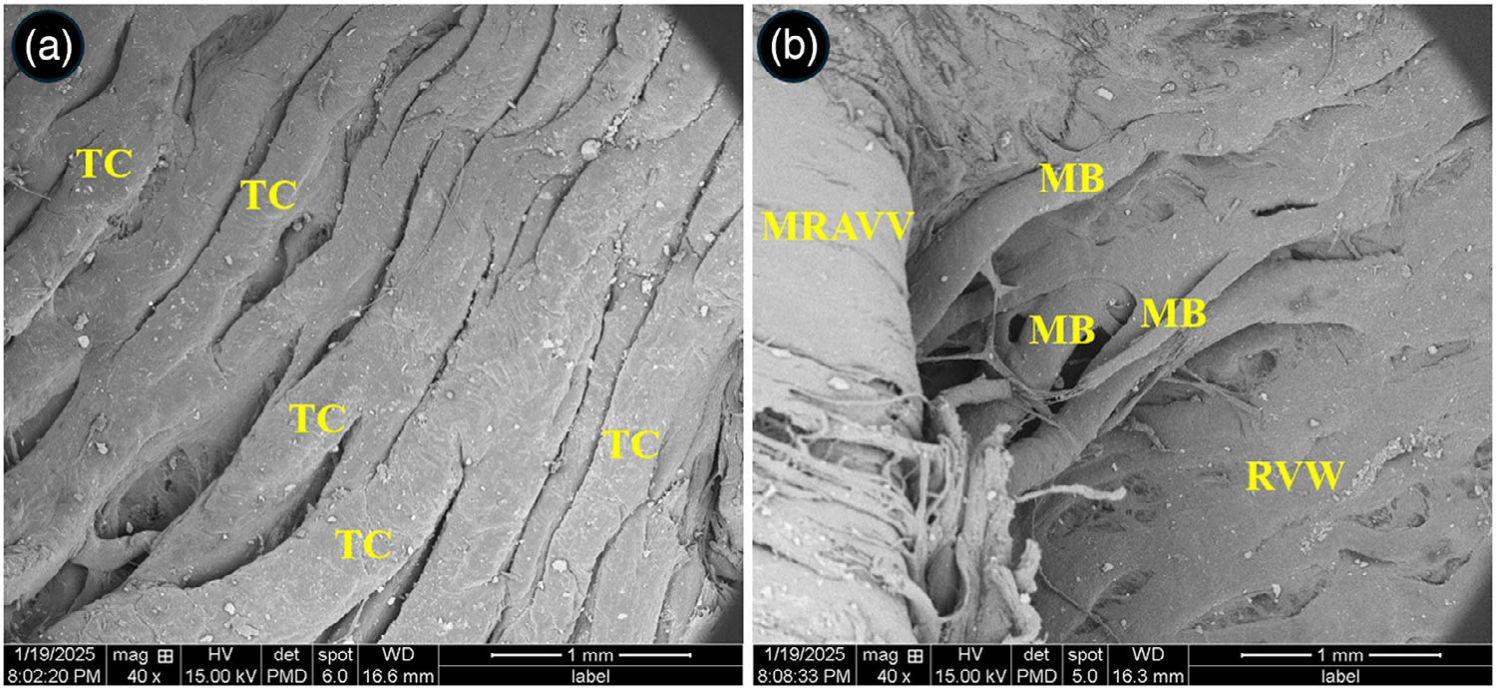
SEM micrograph of the right atrioventricular valve and ventricular walls in the common pheasant heart. (A) shows free wall of the left ventricle with numerous trabeculae carinae (TC), (B) shows muscular right atrioventricular valve (MRAVV) which connected to right ventricular wall (RVW) through several muscular bands (MB).

At the level of SEM, the atria and auricles were composed of parallel arrays of muscles and some fenestrations were seen between them (Figure 6A,B). The chordae tendineae of the left atrioventricular valve showed a branched appearance and each chordae tendineae was composed of three to four narrower cords twisted to each other's and attached to a common papillary muscle (Figure 6C). The surface of cusps had primary and secondary folds (Figure 6D). The endocardial surface of the ventricles was designed by numerous trabeculae carinae (Figure 7A). The right muscular AV valve was attached to the right ventricular free wall by several muscular cords (Figure 7B).

### 3.3 Morphometric Measurements

The results of gross morphometric measurements are presented in Table 1. The mean heart mass was 7.39 ± 0.22 g and the relative heart mass was calculated as 0.27 ± 0.04%. The length and width of the heart were measured as 2.94 ± 0.46 and 2.38 ± 0.15 cm, respectively. The diameters of the aortic arch, left and right brachiocephalic trunks were recorded as 3.32 ± 0.12, 2.2 ± 0.31 and 2.45 ± 0.29 mm. The diameter of left and right pulmonary arteries were recorded as 2.25 ± 0.15 and 2.27 ± 0.33 mm (Table 1).

**TABLE 1:** Morphometric parameter measurements in the heart of the common pheasant (*Phasianus colchicus*) (*n* = 5).

Parameter	Value
Relative heart mass	0.27 ± 0.04
Heart length (cm)	2.94 ± 0.46
Heart width (cm)	2.38 ± 0.15
**Diameter**	
Aorta (mm)	3.32 ± 0.12
Left pulmonary artery (mm)	2.25 ± 0.15
Right pulmonary artery (mm)	2.27 ± 0.33
Left brachiocephalic trunk (mm)	2.2 ± 0.31
Right brachiocephalic trunk (mm)	2.45 ± 0.29

### 3.4 Histometric Measurements

The results of the histometric measurements are presented in Tables 2 and 3. The LVW at middle and apical regions was thicker than RVW four times and three times, respectively (4.55 ± 0.53 vs. 1.11 ± 0.3 mm and 1.61 ± 0.3 vs. 0.52 ± 0.08 mm, respectively, *p* < 0.05). The basal region of the LVW was significantly (*p* < 0.05) thicker than other regions of the LVW (4.33 ± 0.29). The difference of thickness between the regions of the interventricular septum was not significant (*p* > 0.05). The apical and basal regions of the RVW were thinner than the middle region (*p* < 0.05) (Table 2). Overall, the thickness of the RVW and LVW decreased from the base to apex of the heart. The wall thickness of the aorta, left and right brachiocephalic trunks were 362.12 ± 16.07, 322.12 ± 48.56 and 280.8 ± 26.04, respectively (Table 3). Although the wall thickness and luminal diameter in the left bravhiocephalic trunk and left pulmonary artery were larger than their right counterparts; however, the differences were not significant (*p* > 0.05). These parameters for the aorta and brachiocephalic trunks were significantly higher in comparison to the pulmonary arteries (*p* < 0.05).

**TABLE 2:** Mean thickness of the walls of the ventricles and atria in the common pheasant (*Phasianus colchicus*) (*n* = 5).

	Basal point	Middle point	Apical point
Left ventricular wall (mm)	4.33 ± 0.29^a^	4.55 ± 0.52^a^	1.61 ± 0.3^b^
Right ventricular wall (mm)	0.35 ± 0.01^a^	1.11 ± 0.3 ^b^	0.52 ± 0.08^a^
Interventricular septum (mm)	3.71 ± 1.1^a^	3.65 ± 0.9^a^	3.24 ± 1.21^a^
LVW/RVW	12.37 ± 1.26^a^	4.1 ± 0.84^b^	3.09 ± 0.65^b^
Left atrial wall	242.51 ± 8.74		
Right atrial wall	238.21 ± 12.33		

*Note*: Different superscript letters in the same rows indicate significant differences at the level of *p* < 0.05.

**TABLE 3:** Mean ± mean of wall thickness, lumen diameter and luminal area of the large arteries in the common pheasant (*Phasianus colchicus*) (*n* = 5).

	Wall thickness (µm)	Luminal diameter (µm)	Luminal area (mm^2^)
Aorta	362.12 ± 16.07^a^	853.8 ± 55.63^a^	5.14 ± 0.9^a^
Left brachiocephalic	322.12 ± 48.56^a^	724.65 ± 21.19^a^	5.04 ± 0.32^a^
Right brachiocephalic	280.8 ± 26.04^a^	834.15 ± 65.81^a^	4.28 ± 0.71^a^
Left pulmonary	131.8 ± 11.98^b^	760.41 ± 35.2^a^	3.61 ± 0.97^b^
Right pulmonary	107.6 ± 15.72^b^	754.83 ± 18.2^a^	3.27 ± 0.55^b^

*Note*: Different letters in the same columns indicate significant differences at the level of *p* < 0.05.

### 3.5 Histological Observations

**FIGURE 8:**
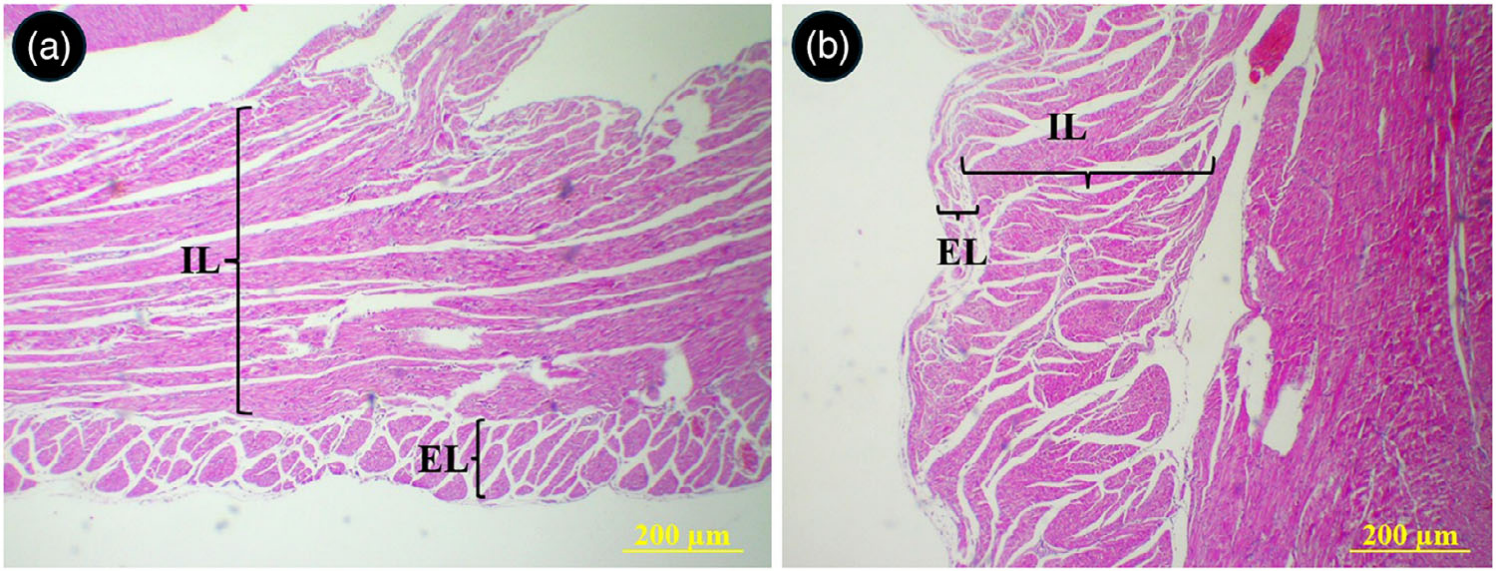
Histological section of the right ventricular wall in the common pheasant heart. Th wall was composed of two district external (EL) and internal (IL) muscular layers. (A) shows right ventricular I transverse section, (B) shows right ventricular wall in longitudinal section (H&E staining).

**FIGURE 9:**
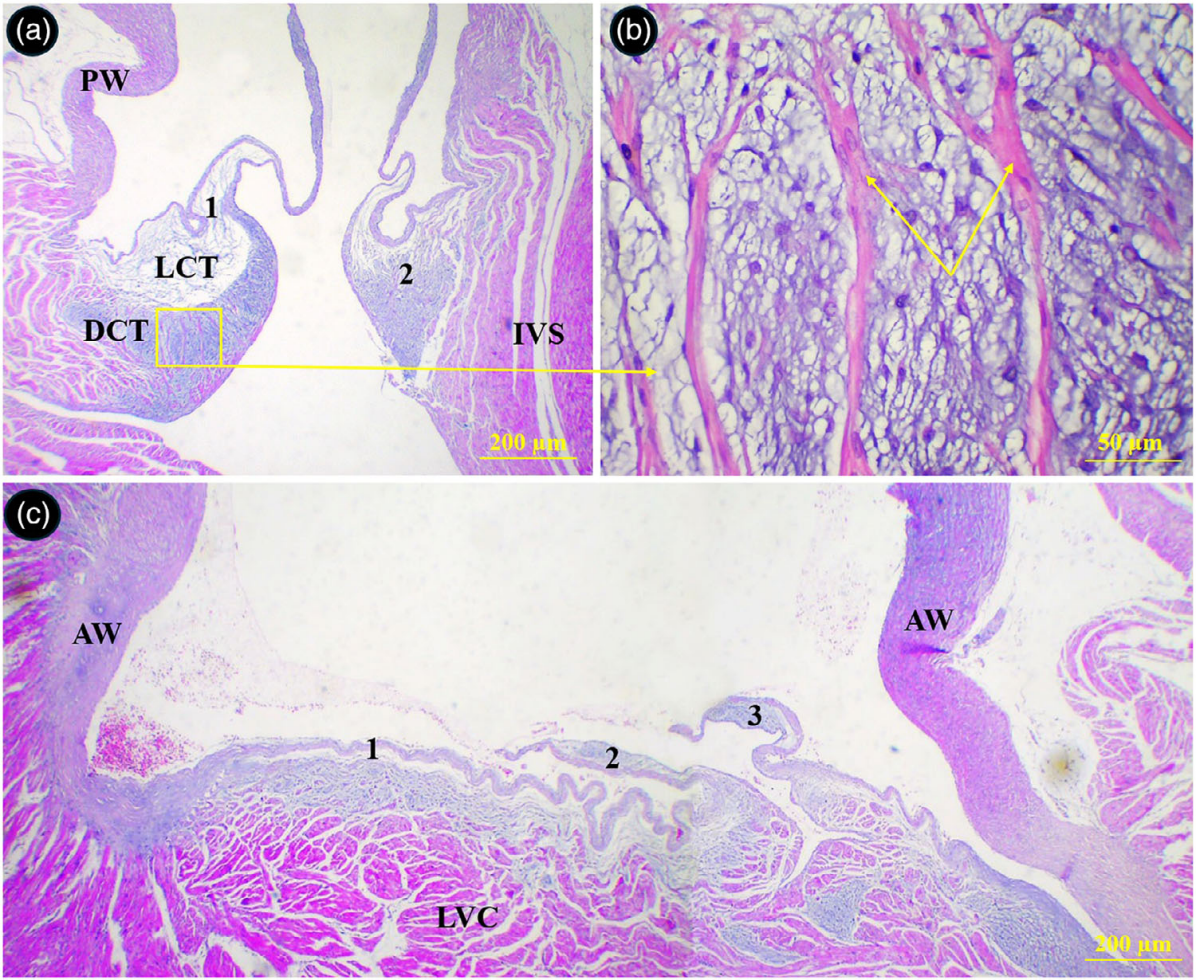
Histological section of the aortic and pulmonary semilunar valves in the common pheasant heart. (A) Shows two cusps of the pulmonary semilunar valves which were composed of loose (LCT) and dense (DCT) connective tissues. (B) Shows extension of the muscular fibres into the cusps (arrows) selected in A at higher magnification. (C) Shows three cusps of the aortic semilunar valve at the base of the aorta. (1) right cusp, (2) left cusp, (3) intermediate cusp, PW: pulmonary artery wall, IVS: interventricular septum, AW: aortic wall, LVC: left ventricular cardiomyocytes (H&E staining).

**FIGURE 10:**
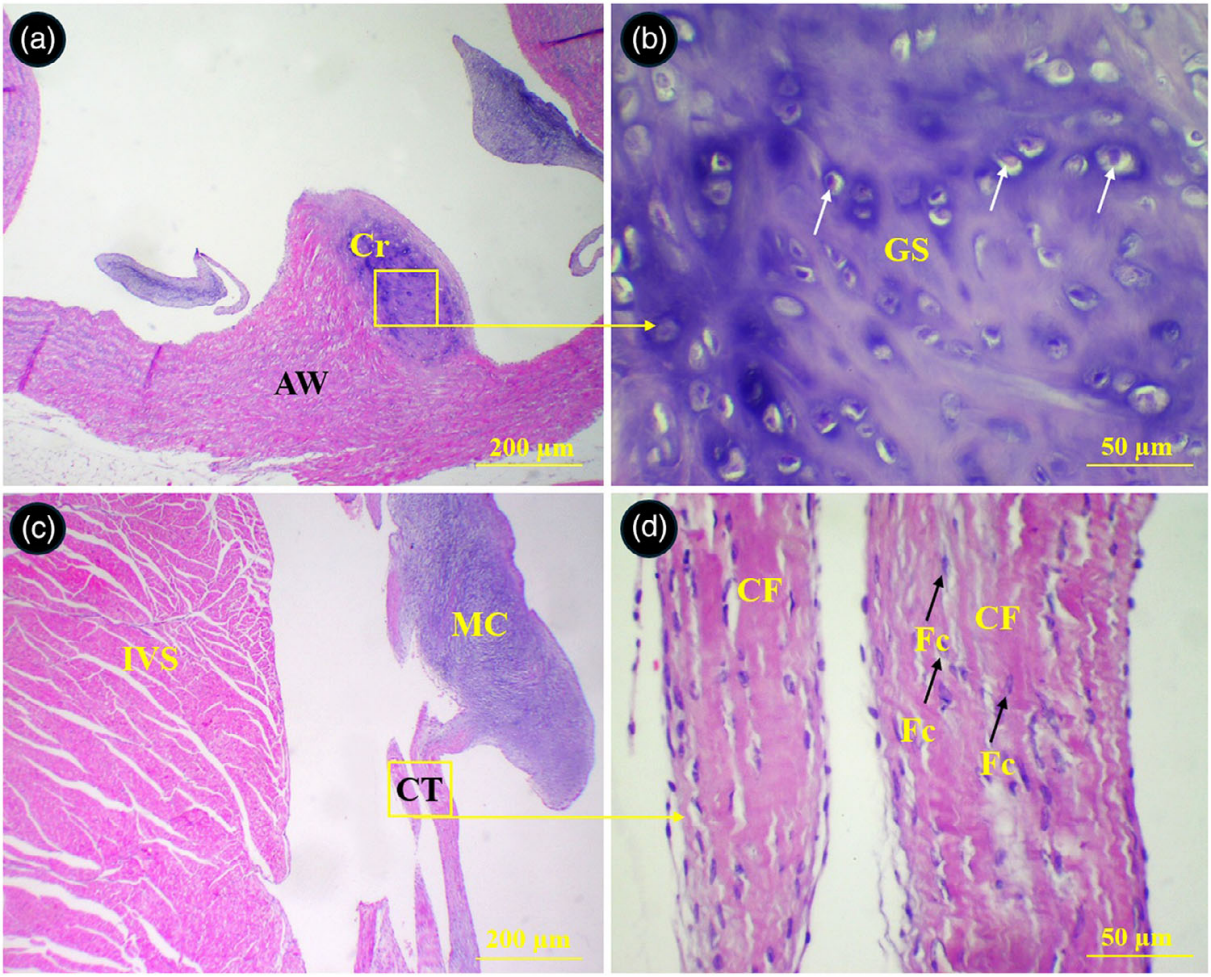
Histological section of the aorta and left atrioventricular valves in the common pheasant heart. (A) Shows a piece of cartilage (Cr) incorporated into the wall of the aorta, (B) shows selected box in A at higher magnification, (C) shows two chorda tendineae connected to a cusp, (D) shows selected box in C at higher magnification. GS: ground substance, arrows: chondrocytes, IVS: interventricular septum, CT: chordae tendineae, MC: mitral cusp, CF: collagen fibres, Fc: fibrocyte (H&E staining).

The parietal wall of the right ventricle was composed of two distinct muscular layers. The external layer was thin with longitudinally oriented muscular fibres. The internal layer was much thicker and constituted of several bundles of transversely oriented muscular fibres (Figure 8). The cusps of semilunar valves at the base of the aorta and pulmonary trunks were composed of both loose and dense connective tissues and their surfaces were covered by a thin simple squamous epithelium. The upper portion of the cusps in right semilunar valve was made of a dense connective tissue which was penetrated by numerous muscular fibres from ventricular wall. The lower portion of cusps was made of a loose connective tissue and continued by chordae tendineae (Figure 9). A piece of cartilaginous tissue was present at the junction of the aorta and its semilunar valve (Figure 10A,B). The chorda tendinea was composed of bundles of collagen fibres and fibrocytes (Figure 10C,D).

**FIGURE 11:**
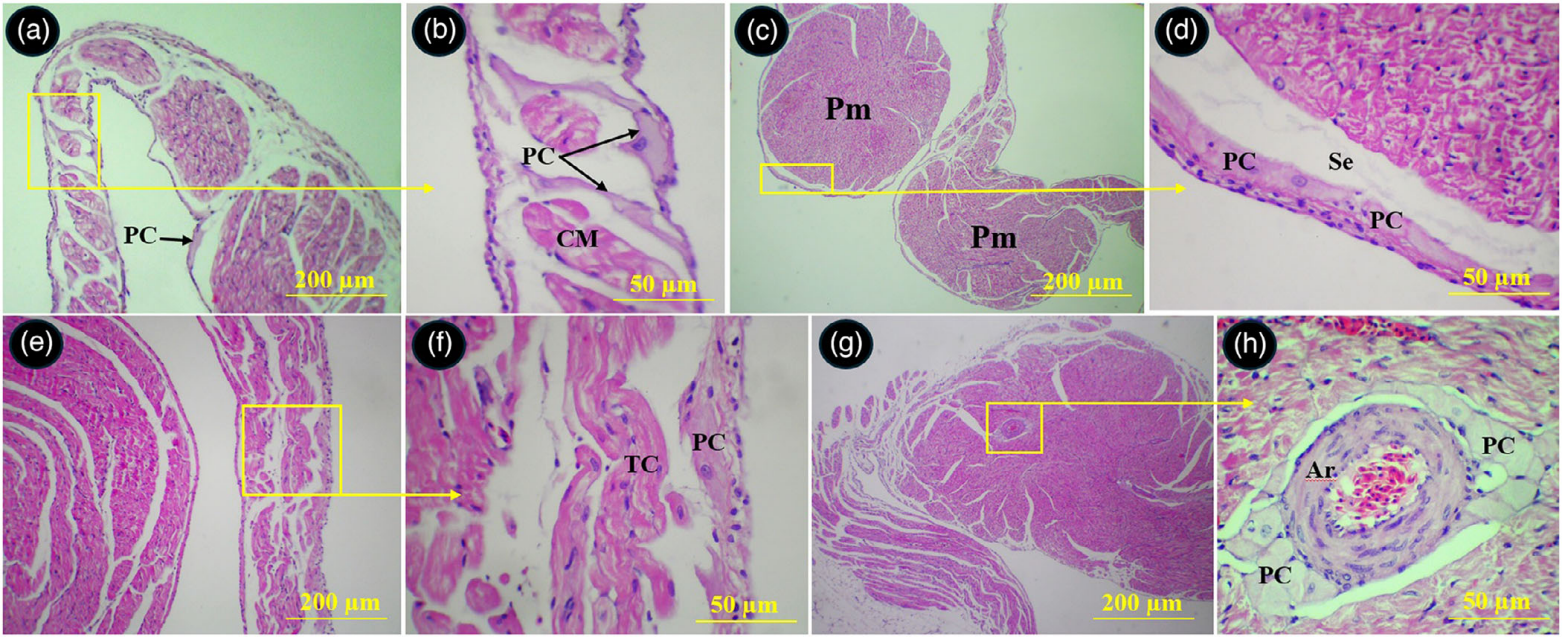
Distribution of the Purkinje fibres in different parts of the atria in common pheasant heart. (A) shows left atrial wall, (B) shows Purkinje fibres intermingled with bundles of cardiomyocytes (selected box in A at higher magnification), (C) shows two pectinate muscles in the right auricular wall, (D) shows Purkinje fibres in the subendocardial space (selected box in C at higher magnification), (E) shows right atrial wall, (F) shows Purkinje fibres in the subepicardial space (selected box in E at higher magnification), (G) shows a pectinate muscle in left auricular wall, (H) shows perivascular Purkinje fibres in the pectinate muscle (selected box in G at higher magnification). PC: Purkinje fibre, Pm: pectinate muscle, Se: subendocardial space, CM: cardiomyocyte, TC: transitional cells, Ar: arteriole (H&E staining).

**FIGURE 12:**
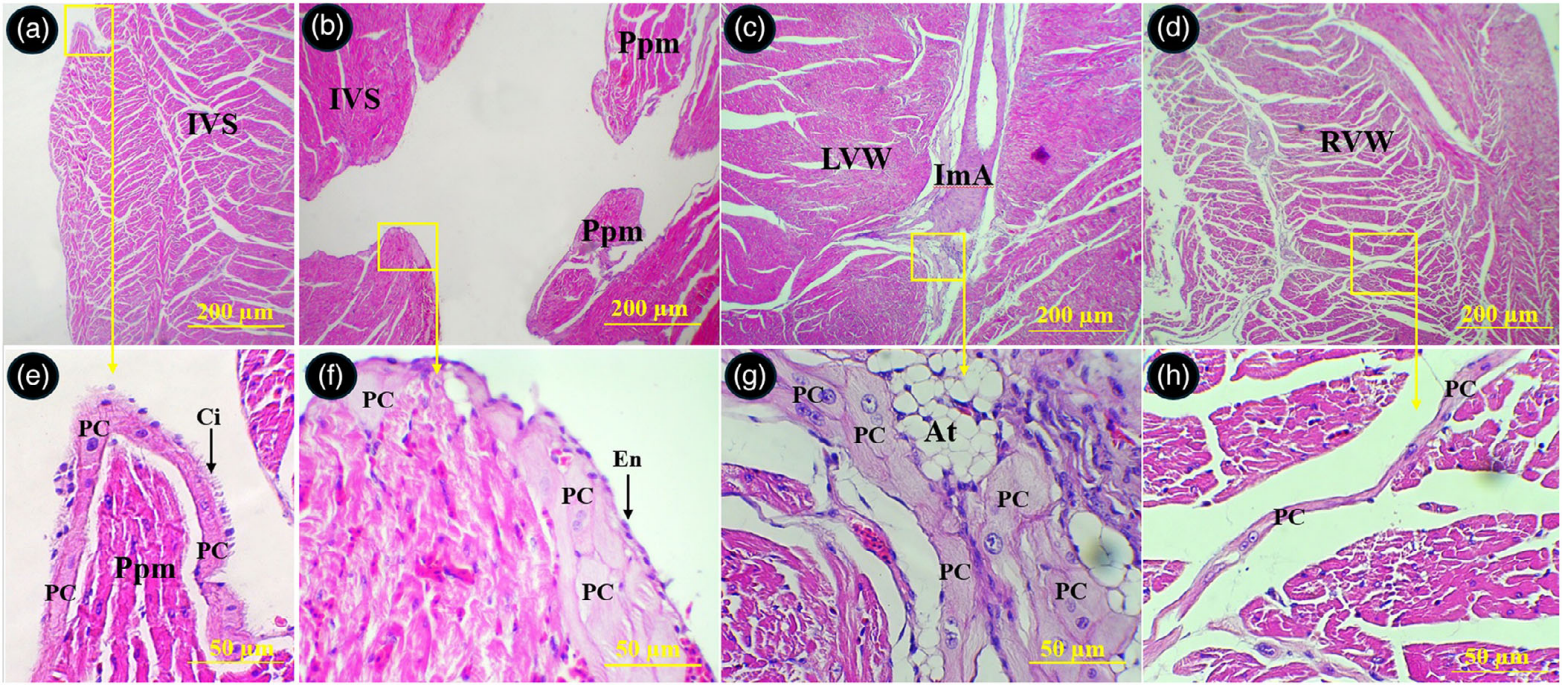
Distribution of the Purkinje fibres in different parts of the ventricles in common pheasant heart. (A) Shows interventricular septum, (B) shows papillary muscle in the left ventricular lumen, (C) shows left ventricular wall, (D) shows right ventricular wall, (E), (F), (G) and (H) show selected boxed in A, B, C and D, respectively at higher magnification. PC: Purkinje fibre, En: endothelium, Ppm: papillary muscle, ImA: intramural arteriole, IVS: interventricular septum, RVW: right ventricular wall, LVW: left ventricular wall, Ci: cilia, At: adipose tissue (H&E staining).

The Purkinje cells of the conductive system were widely distributed beneath the epicardium and endocardium of the atria, under the endocardium of the right and left ventricles, between the myocardium of the ventricular walls and interventricular septum and around arteries in the myocardium. The Purkinje fibres exhibited a pale pink cytoplasm with one or two round centrally located euchromatic nucleus. The external surface of their membrane was equipped with cilia or microvilli like processes (Figures 11 and 12).

### 3.6 Immunohistochemical Observations

**FIGURE 13:**
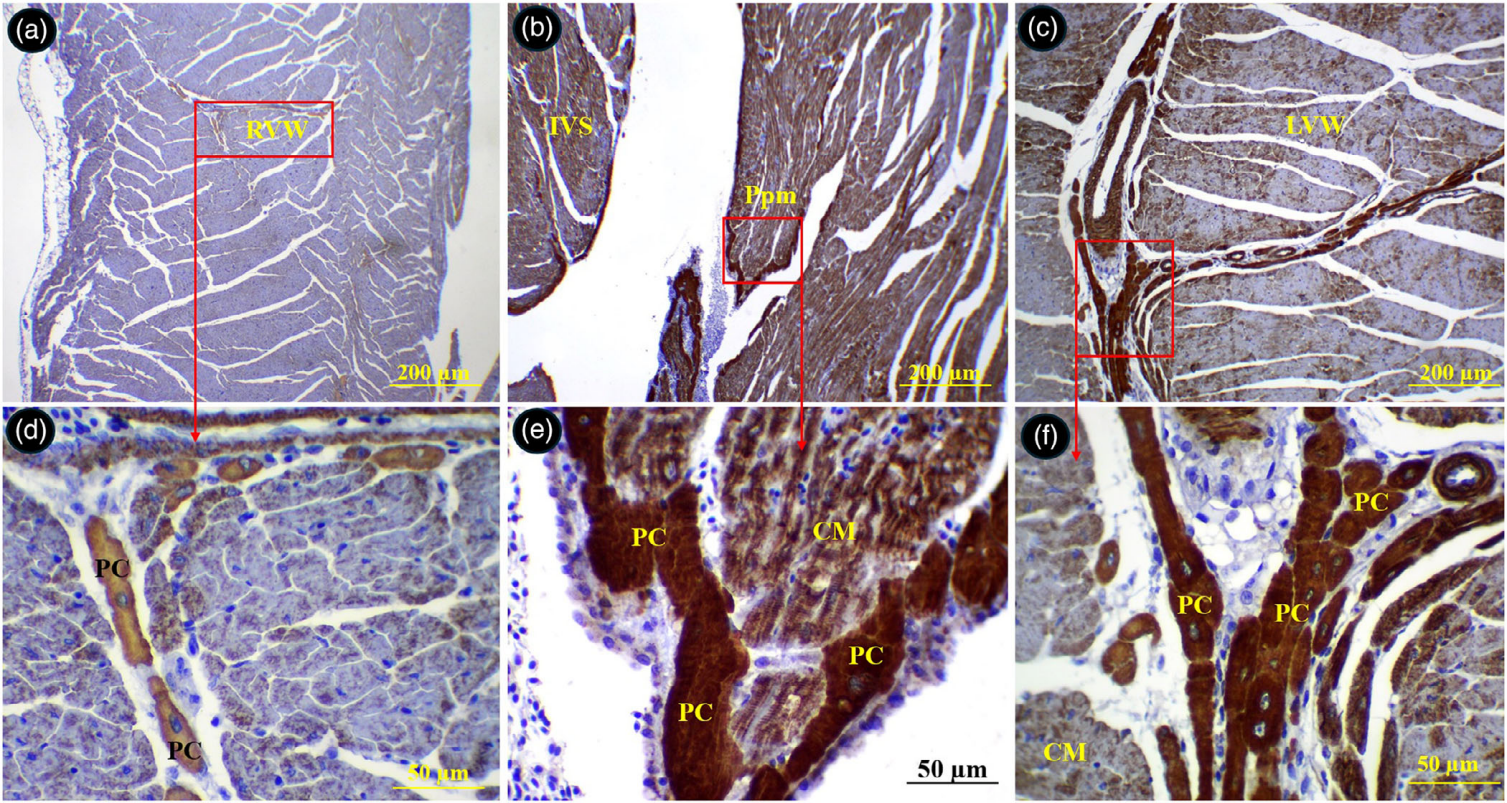
Immunostaining for desmin in different parts of the common pheasant heart. (A) Shows right ventricular wall, (B) shows papillary muscle in the left ventricular lumen, (C) shows left ventricular wall, (D), (E) and (F) show selected boxed in A, B and C, respectively at higher magnification. PC: Purkinje fibre, Ppm: papillary muscle, IVS: interventricular septum, RVW: right ventricular wall, LVW: left ventricular wall (H&E staining).

**FIGURE 14:**
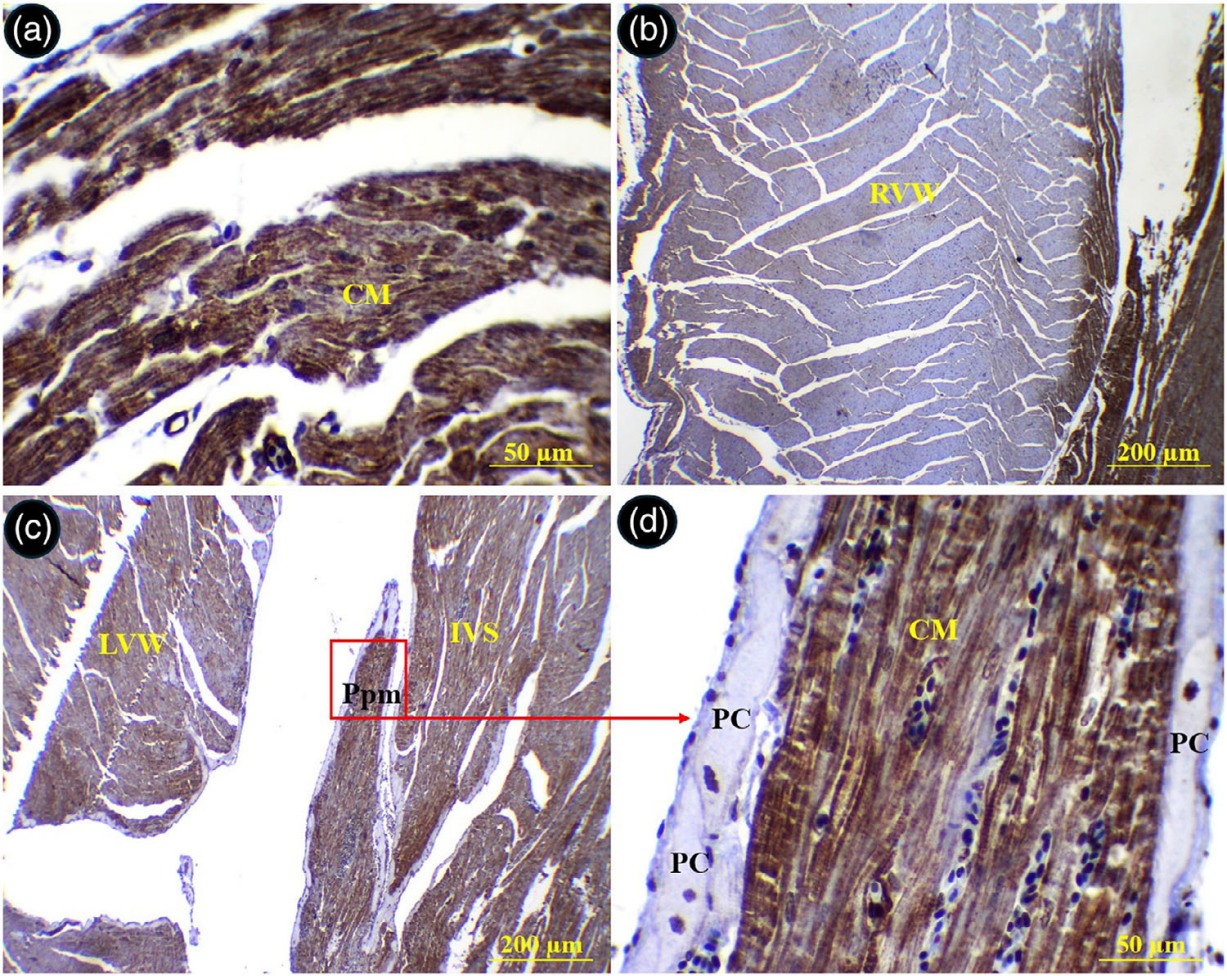
Immunostaining for α‐SMA in different parts of the common pheasant heart. (A) Shows left atrial wall, (B) shows right ventricular wall, (C) shows papillary muscle in the left ventricular lumen, (D) show selected boxed in C at higher magnification. PC: Purkinje fibre, Ppm: papillary muscle, IVS: interventricular septum, RVW: right ventricular wall, LVW: left ventricular wall (H&E staining).

Immunohistochemical staining for desmin showed that the right ventricle wall was negative for this marker; however, the left ventricle and interventricular septum were positive. The Purkinje fibres demonstrated a strong immune‐positive reaction for desmin (Figure 13). The sub‐endocardial Purkinje fibres exhibited stronger reaction as compared to the intramural Purkinje fibres. The parietal walls of the right and left ventricles were negative for α‐SMA, while the wall of the atria and interventricular septum exhibited a positive response to α‐SMA. The Purkinje fibres were immune‐negative for α‐SMA (Figure 14).

## 4 Discussion

The current study elucidated the anatomical and structural characteristics of the heart and its associated great arteries in the common pheasant, thereby providing valuable insights into avian cardiovascular morphology. The observed heart weight in the common pheasant reflects adherence to allometric scaling principles, which posit a predictable relationship between heart mass and overall body mass across species; this suggests that the common pheasant's cardiovascular system is appropriately sized for its metabolic demands and physiological functions.

Although the avian heart is in the cranial part of the thoracoabdominal cavity and is embedded by right and left lobes of the liver, its exact topography can be varied based on the length of the heart and its orientation (Nickel et al. 1977; Getty 1975; King and Mclelland 1984). The location of the common pheasant heart, nestled between the first and fourth ribs, alongside an acute orientation to the sternum. The heart was reported with an orientation parallel to the sternum in pigeon and a more oblique orientation in fowl. Accordingly, the pigeon heart extended from second intercostal space to the sixth intercostal space, however, fowl heat was described to extend between the first and third intercostal spaces (Elewa et al. 2018).

The presence of sterno‐pericardial and hepato‐pericardial ligaments anchoring the heart to the sternum and liver, respectively, highlights the importance of these structures in maintaining cardiac stability within the thoracic cavity. These ligamentous attachments prevent excessive cardiac movement and torsion, which could otherwise compromise vascular integrity or interfere with cardiac filling and contraction.

The relative heart mass in the common pheasant was 0.27%. This parameter can be varied in a wide range from 0.2% to 2.4% in avian species. There are several factors that can influence the size of the heart including age, activity, seasonal variation, climate and altitude. In some cases, the heart would have the same size in both sexes despite a considerable sexual difference in body weight, so no evidence of a difference in heart size was found between the sexes. Although it seems that the heart weight can be increased with age, there are several studies that reported the relative heart mass of poultry diminished with ages (Al Masri et al. 2017; Hassanzadeh et al. 2005; Tickle et al. 2014). Warm seasons are associated with larger hearts in the same species. In species which live at high altitudes the hearts may be larger than related forms found at lower levels. The relative hart mass can be affected directly by the type of locomotion and flying ability of the bird species. The species with bipedal locomotion have lower relative heart masses compared to the flying birds (Hartman 1955). For instance, in the chicken and pigeon the relative heart mass was reported as 0.44% and 1.0%, respectively (Elewa et al. 2018). However, it was stated by some authors that very little change occurs in the cardiac index in birds kept in cages for a long period and that the size of the heart is a constant characteristic for the species (Sakharova 1946).

The results of measuring the thickness of RVW, LVW and IVS showed that the LVW was four times thicker than RVW at the middle region. This finding is also reported in other avian species like those described in mammalian ones (Robinson and Robinson 2015). This variation in thickness can be attributed to the increased pressure exerted by systemic circulation, requiring more effort for this muscle. In other words, greater thickness is due to the effort of the left ventricle to overcome systemic vascular resistance, unlike the right ventricle and the lower pulmonary vascular resistance (Soares et al. 2010). Furthermore, the difference in the thickness of the ventricles can be attributed to the sequence of the excitation of the ventricles in the avian heart. Avian hearts have a ventricular conduction system enabling rapid activation of both ventricles in the myocardium from apex to base (Kharin et al. 2006).

The middle region of the LVW, RVW and IVS were thicker than basal and apical regions. This finding is consistent with previous study which compared three avian species including common buzzard, Alisterus parrot and budgerigar (Straub et al. 2002). Borges et al. (2021) examined the morphometry of the heart in greater rhea and described that the basal regions of the LVW and RVW were thicker than apical and middles regions (Borges et al. 2021). They justified this finding by the myocardial arrangement since the cardiac muscle fibres fold in this region and, when anchored to the atrioventricular rings, form the heart apex (Mehner and Hartfiel 1983). The results of the present work revealed the thickness of RVW as 0.35 and 0.55 mm in basal and apical regions, respectively. However, V. Prosheva et al. (2019) reported values of 1.3 and 1.2 mm for the basal and apical regions of the right ventricle free wall in the chicken (*Gallus gallus domesticus*). By comparing their reported results for heart length and heart width in chicken (4.41 and 2.2 mm) with our data (29.4 and 23.8 mm), it can be concluded that the heart of the common pheasant is smaller and has thinner ventricular walls as compared to the chicken heart which their body size is in a same range. It is noteworthy that the increase in the left ventricular wall thickness was not due to an increase in the number of cardiomyocytes. It was due to an increase in the size of the cardiomyocytes during postnatal growth of the avian heart as described in 31 different avian species (Anatskaya and Vinogradov 2002). Given that the increase in cell size is coupled with larger diffusion distances for oxygen and nutrients of these cells, this may be a possible limiting factor in heart performance. The arrangement of longitudinally and transversely oriented muscular fibres in the right ventricular free wall of the common pheasant likely contributes to its ability to generate both circumferential and longitudinal shortening during systole, thereby optimizing ventricular ejection fraction.

The presence of the right muscular AV valve is a characteristic feature of the heart in birds and monotreme mammals. However, in marsupial and eutherian mammals the right AV have a fibrous structure with three cusps that are attached to the papillary muscles through chordae tendineae (Jensen et al. 2012). According to the present observation, the right AV in the common pheasant was a large muscular flap attached to the free wall of the right ventricle and had a longitudinal attachment to the interventricular septum. This finding is largely consistent with other avian species. However, regarding the connections of the right AV valve, our observation was slightly different compared to that reported in the chicken heart. The muscular right AV valve in the chicken was described with one attachment to the right ventricular free wall and two attachments (dorsal and ventral) to the interventricular septum (V. Prosheva et al. 2019). However, in the common pheasant, the RAVV had one attachment to the right ventricular free wall and a long vertical attachment to the IVS. Furthermore, the ventral surface of the muscular RAVV was attached to the right ventricular free wall via several muscular cords which are not reported in other avian species.

The aortic wall thickness and its luminal diameter of the common pheasant was measured as 362.12 and 853 µm which are lower than those reported in commercial chickens (900 and 3000 µm, respectively) (Harash et al. 2019). The wall thickness and luminal radius are determinant factors for the ability of the arteries to withstand greater wall tension. The measured values implicate a lower mechanical threshold for wall tension in pheasants relative to that of heavier, genetically engineered commercial chickens, whose aortic architecture adapts to support higher blood volumes and pressures attendant with rapid growth and increased metabolic requirements. According to the Laplace's law (*T* = *P* × *r* / *w*), the wall tension (*T*) of arteries is directly proportional to internal pressure (*P*), the radius of the artery (*r*) and wall thickness (*w*) (Lawence‐Brown et al. 2011). Under this principle, an increase in luminal radius, such as that seen in commercial chickens, imposes a magnified tension load on the artery's wall, necessitating an augmented thickness to maintain structural equilibrium. Conversely, arteries with smaller luminal diameters, like those of the common pheasant, experience relatively reduced wall tension for an equivalent pressure, which partially compensates for the thinner wall. To justify this, commercial broilers, subjected to anthropogenic selection pressures for rapid growth, manifest augmented aortic diameters and thicker walls that bolster resistance to elevated haemodynamic forces inherent to their size and metabolic rate (Harash et al. 2019). In contrast, the common pheasant's vascular dimensions reflect a conserved, possibly basal state optimized for their natural lifestyle and circulatory needs. However, the relatively narrow resilience margin compared to commercial chickens underscores potential vulnerability to hypertensive stress or haemodynamic perturbations beyond baseline physiological ranges (Humphrey 2021).

From an evolutionary perspective, the cardiac architecture observed in the common pheasant reflects its intermediate ecological niche between highly cursorial ground birds and strong fliers. The relatively moderate relative heart mass, thinner ventricular walls compared to commercial chickens and conserved MRAVV morphology align with characteristics found in other galliform birds, suggesting a phylogenetically stable cardiovascular bauplan. Expanding comparative analyses to include additional Phasianidae species and integrating molecular or developmental data would support a deeper understanding of the evolutionary trajectories shaping avian cardiac design.

The Purkinje fibres are one of the most important components of the heart conductive system that transmits the electrical excitation from right and left His bundle to the myocardium (Parto et al. 2013). These fibres have different sizes in various species and are usually larger than cardiomyocytes. However, in rats and mice, Purkinje cells are reported to be very similar to ventricular myocytes even a little smaller (Shimada et al. 1992).

In the present work, the Purkinje fibres have an extensive distribution in the sub‐endocardial, sub‐epicardial spaces of both ventricular and atrial wall as well as peri‐arterial position in the myocardium. This large distribution of the Purkinje system is correlated with the high heart rate characteristic of birds. The close proximity of Purkinje cells to arterial structures suggests a potential interplay between the cardiac conduction system and the coronary vasculature in modulating myocardial perfusion and electrical stability. The distribution of Purkinje fibres within the auricles and atria in the common pheasant heart resembles that described in the pigeon and ostrich (Truex 1961; Parto et al. 2013). Prakash (1956) has stated that the atria of the common Indian fowl are devoid of Purkinje fibres. In our study, they are found in the sub‐endocardial space, within the myocardium in association with blood vessels, and occasionally in the epicardium. Furthermore, the intramural Purkinje fibres in the heart of the common pheasant formed an extensive network between the ventricular myocytes especially in the interventricular septum and left free ventricular wall. Purkinje fibre network in the ventricular myocardium results in a more efficient excitation of the ventricles compared to the absence of intramural Purkinje fibres (Oosthoek et al. 1993).

Our investigation into the Purkinje fibres of the common pheasant heart reveals distinct immunohistochemical properties that can be helpful in discriminating them from the ventricular and atrial cardiomyocytes. The pronounced immunoreactivity of Purkinje fibres to desmin in the common pheasant heart is a notable finding that aligns with the recognized cytoskeletal characteristics of these conductive cells. Desmin, an intermediate filament protein integral to the structural integrity and mechanical stability of muscle cells, exhibits strong expression within Purkinje fibres, discerning them clearly from ventricular and atrial cardiomyocytes (Paulin and Li 2004; Kugler et al. 2018). This differential expression underscores the specialized architecture of Purkinje fibres, designed to withstand the mechanical demands of rapid electrical conduction. This finding is consistent with previous studies that indicate an abundant presence of desmin in the cardiac conduction system of various species, suggesting a conserved role in maintaining conductive cell function (Yamamoto et al. 2011; Capetanaki 2000; Kachinsky et al. 1995). The robustness of the desmin network likely provides the Purkinje fibres with enhanced resilience against mechanical stress, a prerequisite given their role in orchestrating synchronous ventricular contraction. A particularly compelling aspect of our findings is the regional variation in desmin immunoreactivity within the Purkinje fibre network, with the sub‐endocardial fibres exhibiting a more intense desmin reaction compared to intramural fibres. This difference may reflect functional heterogeneity or developmental gradients within the conduction system. The heightened desmin expression in sub‐endocardial tissue could correlate with augmented mechanical or electrical demands in this region, requiring reinforced cytoskeletal support. Such regional variability has been observed in other species and posited to relate to differences in fibre maturity, connectivity or susceptibility to mechanical stress (Kugler et al. 2024). It raises intriguing questions about the plasticity and adaptive mechanisms within the conduction system, suggesting that Purkinje fibres modulate their intermediate filament composition in accordance with local biomechanical environments.

Intriguingly, the complete absence of α‐smooth muscle actin (α‐SMA) expression in common pheasant Purkinje fibres diverges from certain embryonic or pathophysiological contexts where α‐SMA positivity has been documented. α‐SMA is frequently recognized as a marker associated with contractile elements in smooth muscle and myofibroblast differentiation, often implicated in tissue remodelling and fibrosis. While some studies have identified α‐SMA immunoreactivity in Purkinje fibres suggestive of an embryonic‐like or reactive phenotype (Giordano et al. 2024). Furthermore, there was a different expression pattern of α‐SMA in between the atria and ventricles in the present work. The wall of the atria was positive, while the free wall of ventricles was negative. Three α‐muscle actin isoforms are sequentially expressed during in‐vivo cardiac development. α‐SMA is first and transiently expressed, followed by α‐skeletal (α‐SKA) and finally α‐cardiac actin (α‐CAA). In normal myocardium, these isoforms are co‐expressed and the amount of their transcripts has been shown to vary with species, developmental stage, ageing and during pathological situations (Carrier et al. 1992; Schwartz et al. 1992; Winegrad et al. 1990). During in vivo cardiogenesis, α‐SMA marks the onset of cardiomyocyte differentiation, and as development proceeds, it is sequentially replaced by α‐SKA and α‐CAA isoforms (Ruzicka and Schwartz 1988; Woodcock‐Mitchell et al. 1988). The presence of multiple α‐actins appears to provide a diversity in contractile properties that may be related to the physiological demand at the level of the cell and tissue. A mutation in the αCA gene of BALB/c adult mice revealed a correlation between the relative actin isoform content and cardiac function. Further studies are needed to illuminate the expression pattern of actin isoforms in the developing heart of the avian species.

Although the present work focused on structural characterization, correlating these findings with physiological parameters—such as cardiac output, ventricular ejection patterns and conduction velocity—would provide a more comprehensive functional perspective. Many of the observed features, including the prominent Purkinje fibre network and the relative ventricular wall thickness, are consistent with the high metabolic demands of galliform birds. Future studies integrating echocardiographic, electrophysiological or haemodynamic assessments would help clarify how these morphological characteristics translate into species‐specific cardiovascular performance.

A limitation of the present study is the small sample size and the inclusion of only adult males. While similar morphological studies in avian species frequently rely on limited sample numbers due to availability constraints and the labour‐intensive nature of histological and ultrastructural examinations, this restricts the generalizability of the findings. Future studies incorporating females, younger age groups and a larger population would allow assessment of sexual dimorphism, age‐related remodelling and individual variability, thereby enhancing the robustness of anatomical and functional interpretations.

## 5 Conclusion

In conclusion, this study provides a detailed, integrative overview of the common pheasant's cardiac and arterial anatomy, emphasizing its physiological suitability and species‐specific adaptations. Distinct ventricular wall thickness profiles illustrate pressure‐driven morphological specialization. The unique MRAVV structure adds nuance to avian valvular diversity. Vascular measurements align with biomechanical principles, revealing species‐specific constraints and adaptations. Lastly, the comprehensive Purkinje fibre network with distinctive immunohistochemical traits underscores the efficient cardiac conduction system in this species. Collectively, these findings enrich the knowledge base for comparative avian cardiovascular morphology and provide a foundation for further functional and evolutionary studies.

## Author Contributions

**Hadis Ramezani**: software, investigation, funding acquisition. **Nader Goodarzi**: writing – original draft preparation, conceptualization, methodology, supervision. All authors have read and agreed to the published version of the manuscript.

## Funding

The authors have nothing to report.

## Ethics Statement

The study protocol and animal experiments were approved by the Ethics Committee of Razi University, Iran (Approval no: IR.RAZI.AEC.1403.060).

## Conflicts of Interest

The authors declare no conflicts of interest.

## Data Availability

The data that support the findings of this study are available from the corresponding author upon reasonable request.
